# Case Report: Atypical hemolytic uremic syndrome triggered by Rhinovirus/Enterovirus infection with cutaneous thrombotic microangiopathy requiring surgical management

**DOI:** 10.3389/fped.2026.1973640

**Published:** 2026-10-19

**Authors:** Jiayao Shen, Kaixuan Cai, Weijie Kang, Qiao He, Jian Zhang, Lei Yin

**Affiliations:** 1 Department of Nephrology, Shanghai Children’s Medical Center, School of Medicine, Shanghai Jiao Tong University, Shanghai, China; 2 Department of Burns and Plastic Surgery, Shanghai Children’s Medical Center, School of Medicine, Shanghai Jiao Tong University, Shanghai, China; 3 Department of Pathology, Shanghai Children’s Medical Center, School of Medicine, Shanghai Jiao Tong University, Shanghai, China; 4 Department of Critical Care Medicine, Shanghai Children’s Medical Center, School of Medicine, Shanghai Jiao Tong University, Shanghai, China

**Keywords:** atypical hemolytic uremic syndrome, complement dysregulation, cutaneous thrombotic microangiopathy, eculizumab, Rhinovirus/Enterovirus infection, surgical debridement

## Abstract

Atypical hemolytic uremic syndrome (aHUS) represents a complement-mediated thrombotic microangiopathy (TMA) characterized by microangiopathic hemolytic anemia, thrombocytopenia, and end-organ damage, most frequently affecting the kidneys. Although infection is a well-recognized precipitant, extensive skin involvement is uncommon. Here, we describe a 14-year-old boy who developed aHUS following Rhinovirus/Enterovirus infection. He was found to have anemia, thrombocytopenia, acute kidney injury, hematuria, and proteinuria, and peripheral smear examination demonstrated schistocytes. Complement evaluation showed reduced C3 and factor I concentrations, increased soluble C5b-9, and preserved ADAMTS13 activity. Investigations for Shiga toxin-producing Escherichia coli, systemic autoimmune disease, and alternative infectious etiologies were negative. Even with eculizumab, corticosteroids, therapeutic plasma exchange (TPE), and continuous renal replacement therapy (CRRT), his condition progressed to petechiae, ecchymoses, hemorrhagic bullae, and necrotic lesions of the axilla, neck, and back. Since the necrotic areas failed to heal and were accompanied by continued platelet consumption and a heightened risk of infection, surgical debridement and wound closure were undertaken after the platelet count reached 81 × 10^9/L. Histopathologic analysis of the excised tissue confirmed cutaneous thrombotic microangiopathy with microvascular thrombosis. Following the procedure, platelet count, hemoglobin, and complement C3 returned to normal, and the wound healed without complication. At subsequent follow-up, the scars were supple and level, with no sensory deficit or functional limitation. This report expands the range of infectious triggers associated with aHUS and demonstrates that persistent necrotic cutaneous thrombotic microangiopathy may necessitate prompt surgical management in addition to complement inhibition and supportive treatment.

## 1 Introduction

Atypical hemolytic uremic syndrome (aHUS) is a severe form of thrombotic microangiopathy (TMA) caused by dysregulation of the alternative complement pathway (1, 2). Clinically, it is defined by the triad of Coombs-negative microangiopathic hemolytic anemia, thrombocytopenia, and acute kidney injury, often accompanied by hematuria and proteinuria (1, 2). The kidney is the principal target organ, although extrarenal involvement may occur and affect the central nervous system, gastrointestinal tract, heart, and, rarely, the skin (2, 3). Current understanding supports a multihit model: inherited or acquired defects in complement regulation create susceptibility, and a triggering event, most commonly infection, precipitates overt disease (2, 4). Viral respiratory infections have been recognized as important triggers, but Rhinovirus/Enterovirus-associated aHUS remains exceedingly rare (5–7). Cutaneous TMA is also an unusual manifestation and may pose major diagnostic and therapeutic challenges when it progresses to hemorrhagic bullae and necrosis (3, 8). For pediatric immune-mediated inflammatory disease, this clinical phenotype is of particular interest because it reflects the intersection of immune dysregulation, endothelial injury, complement activation, coagulation disturbance, and severe skin involvement. We report a pediatric case of aHUS triggered by Rhinovirus/Enterovirus infection, complicated by severe cutaneous TMA refractory to medical therapy and ultimately requiring surgical debridement and closure.

## 2 Case report

A previously healthy 14-year-old boy was admitted in May 2024 with a 3-day history of vomiting, diarrhea, limb numbness, and weakness. Three weeks before admission, he had developed a paroxysmal dry cough, followed 1 week later by fever ranging from 37.5 to 38.3 °C. Before hospitalization, he had received cefaclor, dextromethorphan, and ambroxol without clear clinical improvement. There was no history of eating suspicious or contaminated food. On admission, he was febrile and hypertensive. Laboratory testing showed hemoglobin 79 g/L, platelet count 94 × 10^9/L, and serum creatinine 175 μmol/L. Inflammatory indices were elevated, with C-reactive protein 122.2 mg/L and procalcitonin 11.27 ng/mL. Respiratory pathogen nucleic acid testing was positive for Rhinovirus/Enterovirus, and chest computed tomography showed an infiltrative lesion in the left upper lobe. Intravenous meropenem was started.

During hospitalization, the peripheral blood smear showed schistocytes, and urinalysis revealed hematuria and proteinuria. To exclude other causes of TMA, stool studies including testing for Shiga toxin-producing Escherichia coli, blood next-generation sequencing, and autoimmune serologic studies were performed, and all were negative. ADAMTS13 activity was within the normal range. Complement evaluation showed markedly decreased C3 and factor I levels together with significantly elevated soluble C5b-9, supporting the diagnosis of complement-mediated TMA. Whole-exome sequencing did not identify a pathogenic variant associated with aHUS. There was no known family history of renal disease, hematologic disorders, autoimmune conditions, or chronic dermatologic disorders in the patient's family. By the third hospital day, the patient's condition had worsened, with progressive acute kidney injury, thrombocytopenia, and ongoing microangiopathic hemolysis. A diagnosis of aHUS was established, and treatment was started with high-dose methylprednisolone pulse therapy, eculizumab, therapeutic plasma exchange (TPE), and continuous renal replacement therapy (CRRT). Additional supportive measures included antihypertensive therapy, intravenous immunoglobulin, and plasma infusion.

Approximately 2 weeks after disease onset, he developed multiple ecchymoses over the trunk, which progressively worsened. Within the following week, scattered hemorrhagic bullae appeared in the left axillary region, posterior neck, and back, then evolved into necrotic lesions (Figure 1). Coagulation testing showed marked prolongation of prothrombin time and activated partial thromboplastin time, with substantial elevation of fibrin degradation products and D-dimer. Low-molecular-weight heparin (LMWH) was introduced, and the wounds were managed with local dressings. The child underwent four sessions of TPE, four doses of eculizumab therapy, and one course of high-dose methylprednisolone pulse therapy, followed by a maintenance regimen of methylprednisolone at a dose of 2 mg/kg with gradual tapering. Renal function recovered; however, hematologic normalization lagged behind, with platelet count remaining 44 × 10^9/L and hemoglobin 72 g/L, while C3 had not yet normalized (Figure 2). The necrotic skin lesions failed to heal after rupture of the hemorrhagic bullae. Given the persistent tissue necrosis, ongoing platelet consumption, and increasing risk of secondary infection, surgical intervention was considered necessary.

**Figure 1:**
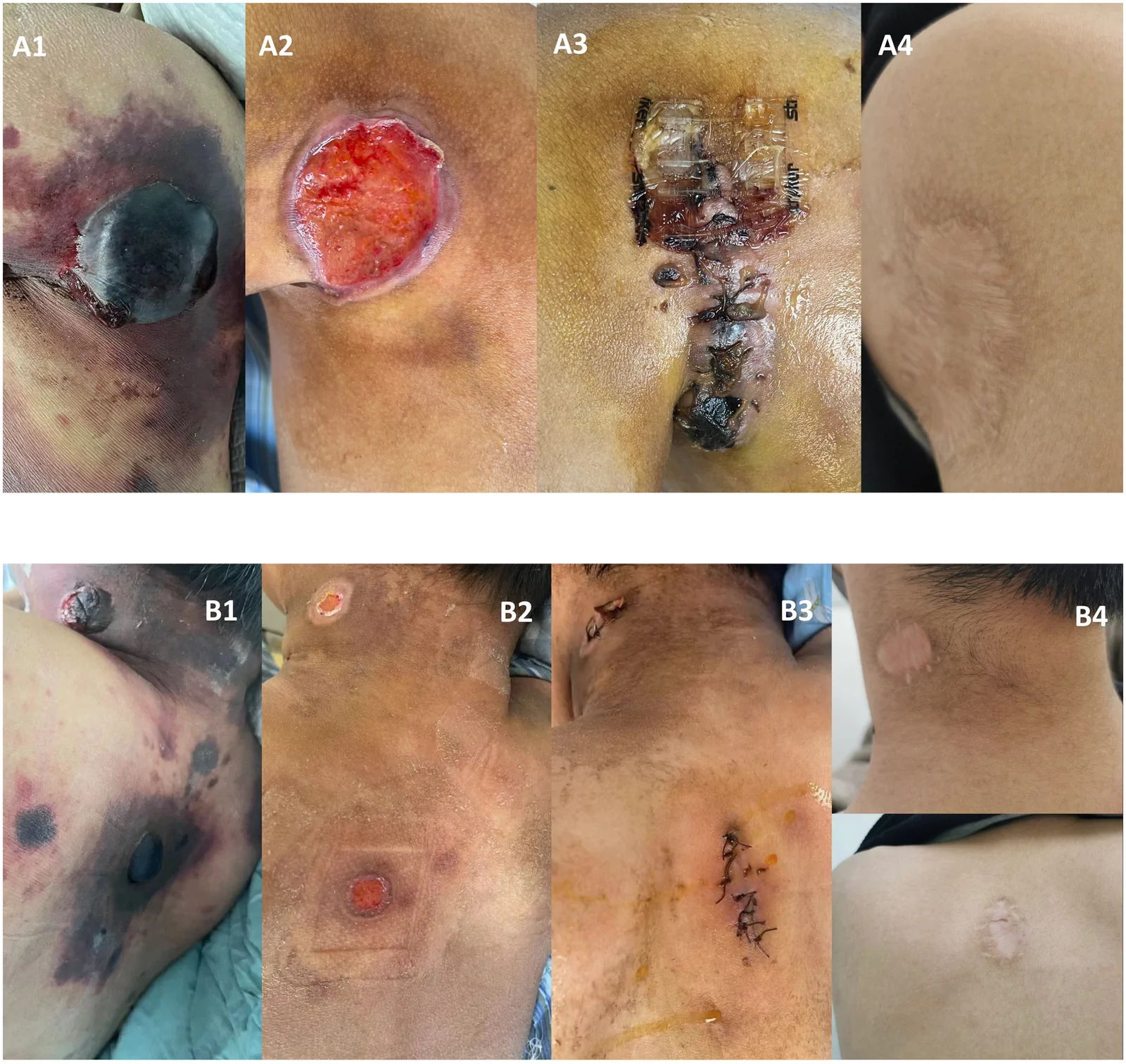
Clinical features of cutaneous thrombotic microangiopathy before and after treatment. Serial clinical photographs show the evolution of hemorrhagic bullous lesions involving the left axilla, nape of the neck, and back. **(A1)** Hemorrhagic bullae in the left axilla; **(A2)** ruptured bullae with cutaneous necrosis; **(A3)** appearance after debridement and suturing; **(A4)** current recovery status. **(B1)** Hemorrhagic bullae on the nape of the neck and back; **(B2)** ruptured bullae with cutaneous necrosis; **(B3)** appearance after debridement and suturing; **(B4)** current recovery status.

**Figure 2:**
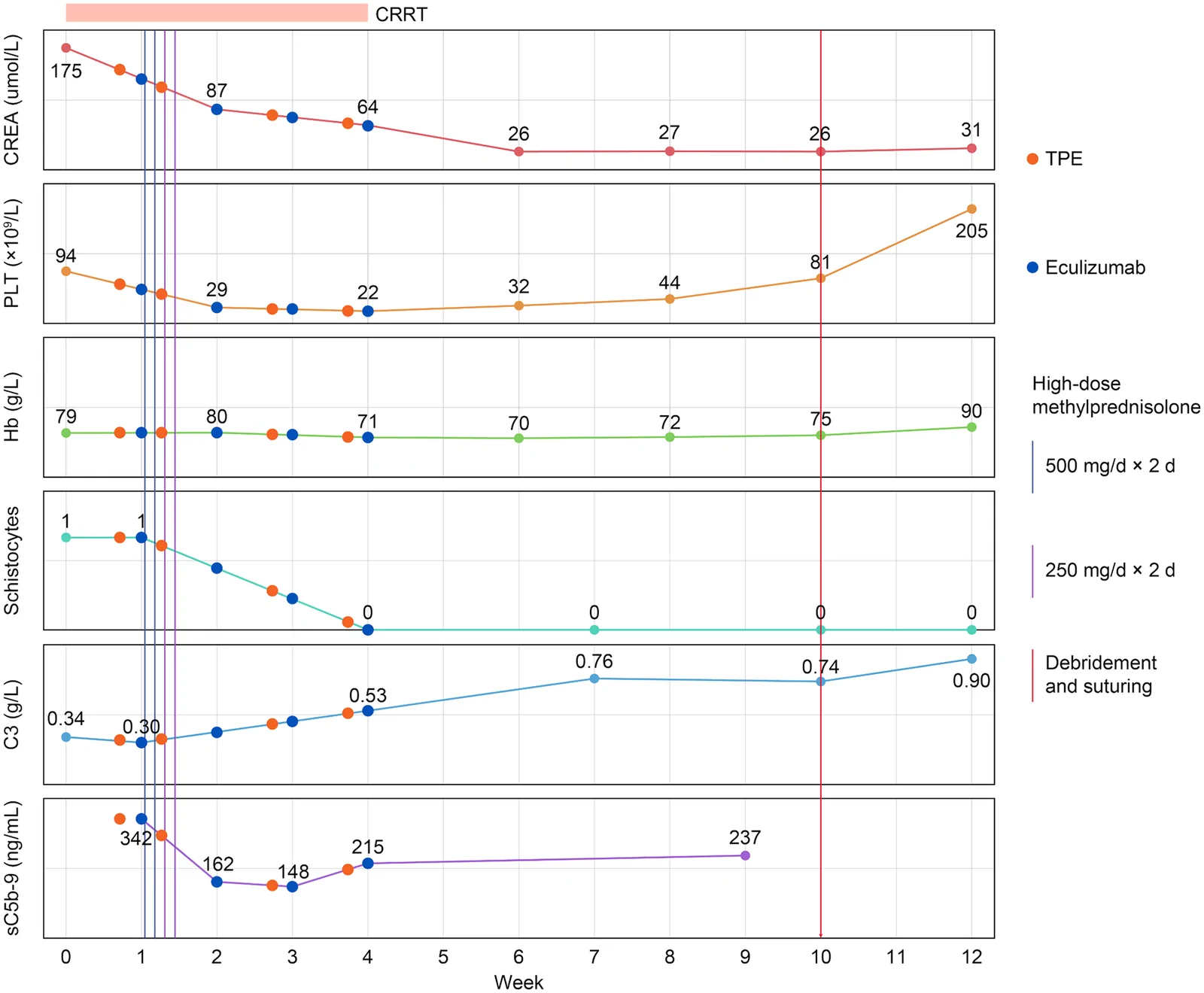
Changes in aHUS-associated clinical parameters throughout the entire treatment course. TPE, therapeutic plasma exchange; CRRT, continuous renal replacement therapy; CREA, creatinine level; PLT, platelet count; Hb, hemoglobin level. Schistocytes: 1 = Presence of schistocytes on peripheral blood smear; 0 = Absence. The reference range for complement C3 is 0.9−1.8 g/L. The reference range for sC5b-9 (soluble C5b-9) is 72−252 ng/mL.

At approximately 10 weeks after admission, once the patient's overall condition had stabilized and the platelet count had risen from 44 × 10^9/L to 81 × 10^9/L, debridement and suturing were performed for the three necrotic wounds involving the left axilla, posterior neck, and back. Total intraoperative blood loss was approximately 60 mL. The patient received 1 unit of packed red blood cells and 1 unit of platelets during the procedure. Hemostasis was achieved with electrocautery and topical epinephrine-soaked gauze. Because of corticosteroid-related skin fragility and dermal atrophy, full-thickness horizontal mattress sutures were used to reduce wound-edge tension and prevent tissue tearing. Histopathological examination of the debrided tissue showed skin necrosis associated with microvascular thrombosis, confirming cutaneous TMA (Figure 3). Postoperative recovery was uneventful. Two weeks after surgery, the platelet count had increased to 205 × 10^9/L, hemoglobin to 90 g/L, and serum C3 had normalized (Figure 2). No postoperative bleeding, wound infection, wound dehiscence, or other unexpected adverse events occurred. Sutures were removed 3 weeks after surgery, and the wounds healed well. The patient adhered well to treatment and follow-up throughout management. At the most recent follow-up, he had returned to regular school attendance and could participate in age-appropriate physical activities without limitation. On physical examination, the scars were soft, flat, and well-matured, with no sensory deficit or functional limitation (Figure 1). Follow-up investigations showed normal 24-hour ambulatory blood pressure, complete blood count, urinalysis, liver and kidney function, electrolyte levels, coagulation profile, and cardiac and abdominal ultrasonography findings. The patient and his legal guardians were satisfied with the functional and cosmetic outcomes. They also appreciated the multidisciplinary care provided by the nephrology, intensive care, pathology, and plastic surgery teams.

**Figure 3:**
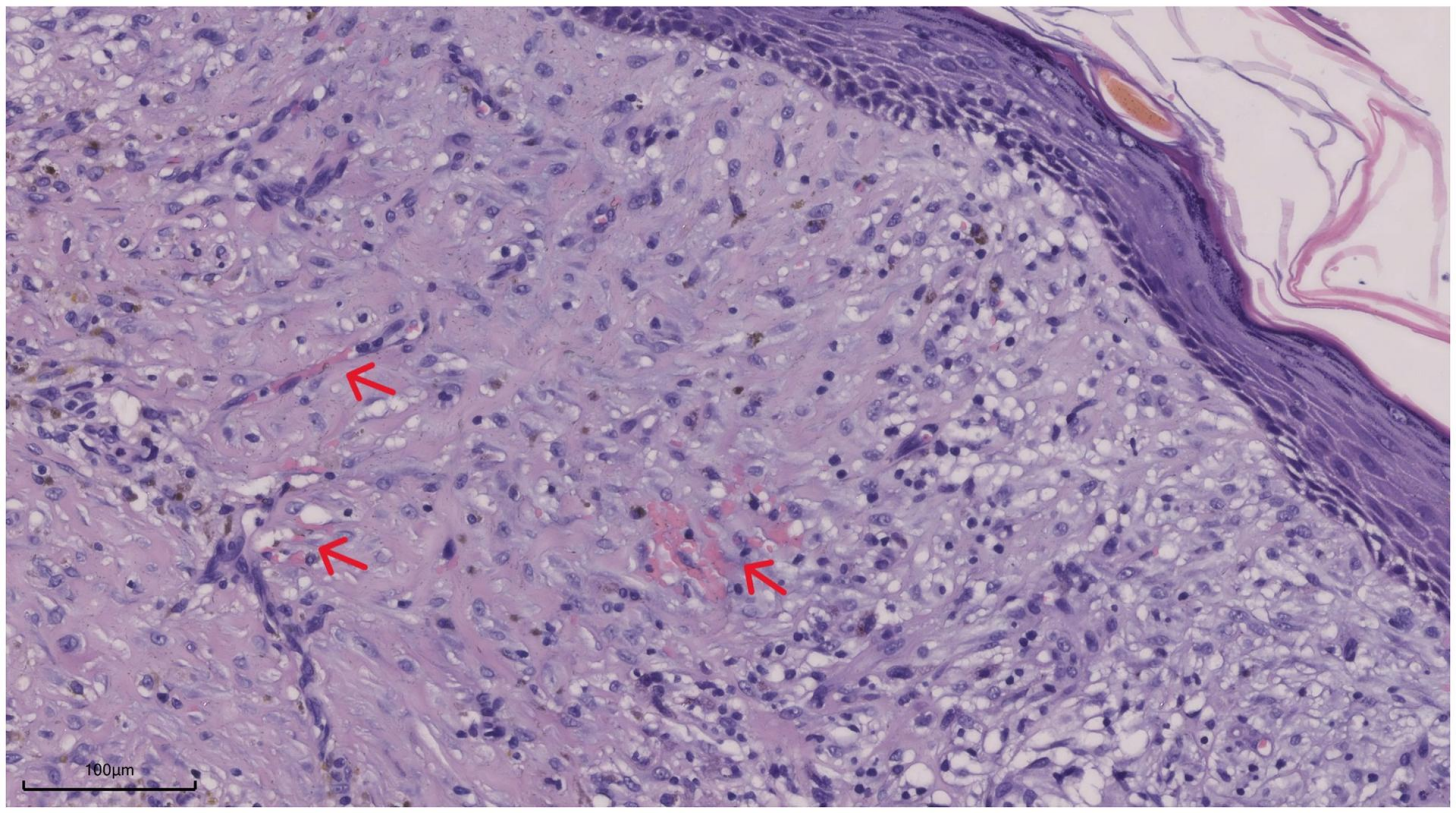
Pathologic evidence of TMA on skin biopsy from the dorsal wound. Light microscopy shows the presence of microthrombi within the dermis (red arrow; Hematoxylin and eosin, ╳200).

## 3 Discussion

This case is notable for several reasons. First, it broadens the spectrum of infectious triggers linked to pediatric aHUS by implicating Rhinovirus/Enterovirus infection. Second, it describes a rare but clinically important dermatologic phenotype of complement-mediated TMA, with hemorrhagic bullae progressing to skin necrosis. Third, it emphasizes the need to combine targeted complement inhibition with appropriately timed surgical management when cutaneous injury persists despite control of the primary systemic process.

Current models view aHUS pathogenesis as a multi-hit process (2, 4, 9). Under physiologic conditions, the alternative pathway remains constitutively active at a low level and depends on strict regulation to prevent autologous endothelial injury (2, 4, 9). This control is mediated by regulatory proteins such as factor H, factor I, and membrane cofactor protein CD46 (2, 4, 9). Inherited or acquired loss of function in these proteins underlies the pathogenesis of aHUS (2, 4, 9). Approximately 60% of patients with aHUS are identified as having complement gene mutations or anti-CFH antibodies (10). Associated complement gene mutations are found in about 5% of healthy individuals (4). Penetrance is low in aHUS families, with an estimated frequency of 10% among individuals carrying the same pathogenic mutation (11). In other words, the mutation confers susceptibility to disease development, not certainty (2). In many cases, a second inflammatory trigger is needed to initiate overt disease, and infection is the most common trigger in childhood (4, 12). Although a range of respiratory pathogens has been implicated, including influenza and SARS-CoV-2, Rhinovirus/Enterovirus has rarely been reported in this setting (5–7). In 2023, Jason Diep et al. (5) reported recurrent aHUS triggered by Rhinovirus/Enterovirus infection in a patient with a CFHR1/CFHR3 gene deletion. In our patient, the temporal relationship between respiratory infection and TMA onset, together with negative testing for Shiga toxin–associated hemolytic uremic syndrome, thrombotic thrombocytopenic purpura, autoimmune disease, and other systemic infections, strongly supports Rhinovirus/Enterovirus as the precipitating trigger. Viral infections have increasingly been recognized as potential amplifiers of dysregulated complement activation in susceptible hosts, which can precipitate endothelial injury and TMA (6, 7).

Skin involvement in aHUS is rare, and severe necrotic lesions are especially uncommon in children (3). In this boy, the skin lesions progressed from petechiae and ecchymoses to hemorrhagic bullae and, ultimately, necrosis. Histopathology showed microvascular thrombosis, confirming cutaneous TMA rather than nonspecific inflammatory blistering or infection-related necrosis. The complement findings were especially informative. Reduced C3 and factor I, together with elevated soluble C5b-9, indicated ongoing alternative pathway consumption and terminal complement activation (4, 9). These biomarkers were diagnostically valuable and were also consistent with the patient's systemic and cutaneous manifestations. Excessive generation of C3b in the setting of inadequate regulation promotes formation of C3 and C5 convertases, culminating in membrane attack complex deposition on endothelial surfaces (4, 9). This leads to endothelial swelling, loss of antithrombotic integrity, platelet activation, and microvascular thrombosis (2, 4). C5a can also induce tissue factor expression on neutrophils and endothelial cells, linking complement activation to coagulation and amplifying thrombogenesis (13). This immune-thrombotic cross-talk likely contributed to the dramatic cutaneous phenotype observed in this patient (Figure 4).

**Figure 4:**
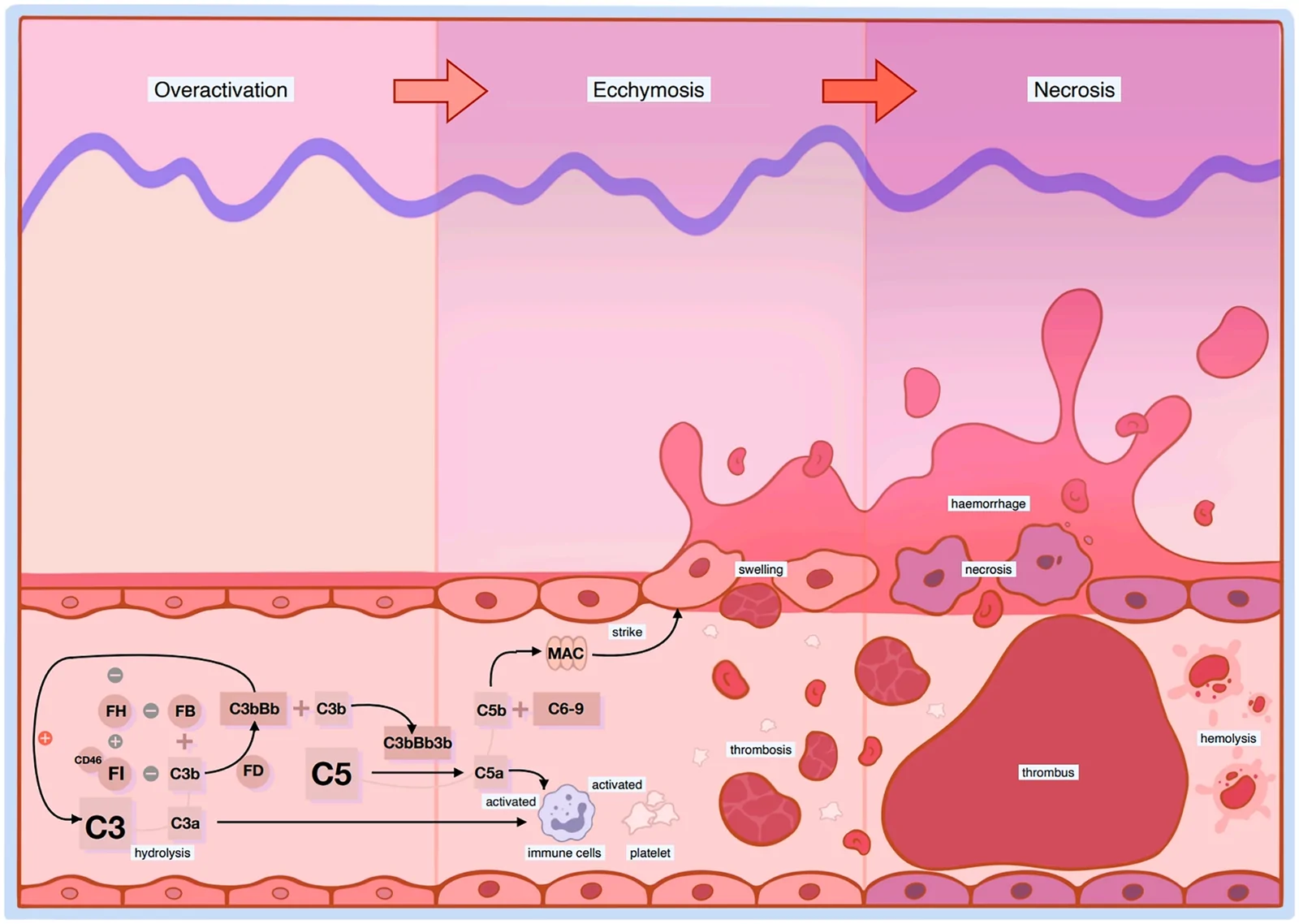
Pathophysiology of subcutaneous microvascular occlusion and secondary skin necrosis mediated by dysregulated overactivation of the alternative complement pathway. FB, factor B; FD, factor D; FH, factor H; FI, factor I; C3bBb, C3 convertase; C3bBb3b, C5 convertase; MAC, the membrane attack complex.

From a therapeutic perspective, this case reinforces the central role of complement inhibition in aHUS. Before biologic therapy became available, TPE was the main treatment option, intended to remove pathogenic factors and replenish complement regulators (2, 14). Eculizumab changed outcomes by specifically blocking C5 cleavage and preventing terminal complement complex formation (14, 15). Early administration is associated with improved renal recovery and better long-term outcomes (14, 15). In our patient, eculizumab was introduced promptly after diagnostic clarification and was followed by improvement in renal function. CRRT, TPE, corticosteroids, and intravenous immunoglobulin (IVIg) were used as adjunctive therapies in the setting of severe multisystem disease. Corticosteroids and IVIg are not primary treatments for complement-mediated aHUS, but in complex inflammatory states they may provide ancillary benefit by modulating systemic immune activation (8). LMWH was also used to address the cutaneous thrombotic component. Even so, despite aggressive medical therapy and stabilization of systemic disease, the necrotic lesions did not heal. Persistent areas of necrotic skin may act as ongoing sites of inflammation, platelet consumption, impaired tissue repair, and microbial colonization (8). In our patient, the failure of wound healing appeared to parallel persistent hematologic abnormalities, particularly thrombocytopenia and anemia.

Surgical management was therefore necessary. In children with atypical hemolytic uremic syndrome, however, the hemorrhagic and thrombotic features require meticulous planning and careful perioperative management, especially with respect to the timing and method of surgery, as well as coagulation and bleeding control. A low platelet count increases bleeding risk and is considered a contraindication to surgery. According to commonly accepted perioperative practice recommendations, invasive procedures should be performed when the platelet count exceeds 50 × 10^9/L, and high-risk operations such as cardiac and neurosurgical procedures require more than 100 × 10^9/L (16). Available evidence suggests that LMWH has not been associated with a clearly increased risk of bleeding complications in dermatologic surgery (17). Out of an abundance of caution and in line with American Society of Regional Anesthesia and Pain Medicine (ASRA) recommendations for perioperative anticoagulant management, we discontinued LMWH 12 h before surgery (18, 19). Other hemostatic precautions included blood product support, meticulous electrosurgical hemostasis, low-tension closure, compression dressings, and immobilization. The subsequent rapid improvement in platelet count and complement normalization after debridement suggests that removal of devitalized tissue may have helped interrupt ongoing local consumption and inflammatory activation, although a direct causal relationship cannot be definitively established in a single case.

Several limitations should be acknowledged. This was a single case report, and definitive causality between Rhinovirus/Enterovirus infection and aHUS cannot be established. The presumed trigger is supported mainly by temporal association and exclusion of other major etiologies. Secondly, although complement testing supported alternative pathway dysregulation, no pathogenic aHUS-associated variant was identified on whole-exome sequencing, and current genetic testing may not detect all clinically relevant complement abnormalities. Finally, multiple therapies were administered in parallel, making it difficult to determine the precise contribution of each intervention.

## 4 Conclusions

This case has broader implications for pediatric immune-mediated inflammatory diseases in dermatology. It shows that dysregulated innate immunity, specifically complement overactivation, can present with striking dermatologic injury in a child. Severe cutaneous thrombotic microangiopathy with hemorrhagic bullae and necrosis is rare, but it may complicate the disease course and contribute to persistent platelet consumption and infection risk. Non-healing necrotic cutaneous TMA should not be viewed solely as a passive consequence of systemic disease. Rather, it may become an active driver of persistent morbidity. In such circumstances, well-timed surgery can be an effective adjunct to complement inhibition and supportive therapy. Comprehensive risk assessment and management of both coagulation and bleeding are vital throughout the perioperative process.

## Data Availability

The original contributions presented in the study are included in the article/Supplementary Material, further inquiries can be directed to the corresponding author.
